# From 60% to 5% in 12 Weeks: A Trastuzumab-Induced Left Ventricular Ejection Fraction Drop

**DOI:** 10.7759/cureus.59172

**Published:** 2024-04-27

**Authors:** Qianjun Pan, Rui Zhao, Suzette Graham-Hill

**Affiliations:** 1 Internal Medicine, SUNY (State University of New York) Downstate Health Sciences University, Brooklyn, USA; 2 Internal Medicine, John H. Stroger, Jr. Hospital of Cook County, Chicago, USA; 3 Cardiology, Kings County Hospital Center, Brooklyn, USA

**Keywords:** congestive heart failure, breast cancer, anthracycline, trastuzumab, cardiotoxicity

## Abstract

Trastuzumab is the first-line therapy for human epidermal growth factor receptor 2 (HER2)-positive breast cancer. However, trastuzumab is associated with cardiotoxicity. It manifests with an asymptomatic reduction of left ventricular ejection fraction (LVEF) and is reversible after discontinuation. Trastuzumab-induced new-onset acute decompensated heart failure is rare (0.5%).

We report a case of a 54-year-old woman who received anthracycline (idarubicin, accumulated dose 400 mg/m^2^ doxorubicin equivalent) for her acute promyelocytic leukocyte 10 years ago, had no relevant comorbidities or other pre-existing cardiovascular diseases, had maintained normal cardiac function, presenting with new-onset dyspnea at rest and bilateral lower extremities swelling 12 weeks after receiving trastuzumab induction chemotherapy for her newly diagnosed early stage HER2-positive breast cancer. Chest X-ray showed severe pulmonary edema. Echocardiography revealed diffuse left ventricular hypokinesis with LVEF 5%. After other possible etiology of cardiomyopathy, including ischemia, infection, substance, or radiation, were excluded by extensive cardiomyopathy workup, a diagnosis of trastuzumab-induced cardiotoxicity was established. Trastuzumab was discontinued, and the patient's symptom was improved with furosemide. Guildline-directed medical therapy was gradually maximized over three months. Repeat transthoracic echocardiography (TTE) at one-year follow-up after the initial diagnosis shows LVEF 33%, and the patient was referred to an advanced heart failure clinic.

This case report demonstrated a rare catastrophic cardiac toxicity effect of trastuzumab and its potential association with remote exposure to anthracycline. Studies have investigated the cardiotoxicity in the concurrent use of trastuzumab and anthracycline therapy. However, how trastuzumab affected patients who were exposed to anthracycline for more than eight years had remained unreported. To our knowledge, no previous detailed case report has described the same clinical scenario as in this case. The case also demonstrates the limitation of the commonly used cardio-oncology cardiovascular risk assessment tool and highlights the importance of individualized cardiovascular risk stratification when deciding on chemotherapy plans.

## Introduction

Trastuzumab improves overall survival and reduces the risk of recurrent disease in early-stage human epidermal growth factor receptor 2 (HER2)-positive breast cancer by inhibiting HER2 receptors. Trastuzumab is associated with dose-independent type II cardiotoxicity, which leads to contractile elements and mitochondrial dysfunction without significant apparent ultrastructural abnormalities [1]. Unlike anthracycline-induced cardiotoxicity (type I), which is characterized by irreversible myocardial damage due to cumulative dose and causes permanent damage and clinical cardiac dysfunction, trastuzumab-induced cardiac dysfunction mainly presents as an asymptomatic reduction of left ventricular ejection fraction (LVEF) and is reversible in most patients after trastuzumab is discontinued. Developing into clinical heart failure is rare: the reported incidence of heart failure with New York Heart Association (NYHA) class III or IV is 0.5-5% [2]. Anthracycline and trastuzumab use concomitantly increases the risk for cardiac toxicity. However, the clinically significant decline in LVEF usually happens in patients with pre-existing cardiovascular risk factors. According to the Herceptin Adjuvant (HERA) trial, a three-month interval between concomitant use is considered cardiac safe, with a reported incidence of heart failure of 0.6% [3]. Here, we report a rare case of an early stage breast cancer in a woman without pre-existing cardiovascular risk factors who was exposed to anthracycline 10 years ago and had maintained normal cardiac function and developed a dramatic LVEF drop from 60% to 5% within 12 weeks of trastuzumab therapy.

## Case presentation

In April 2012, a 44-year-old woman presented to the Kings County Hospital in Brooklyn complaining of sudden-onset left-hand weakness and unexplained ecchymoses. The patient was previously in good health with no comorbidities or taking any medications. A diagnosis of acute promyelocytic leukemia was confirmed by bone marrow biopsy. The patient received a PHEMA regimen (Tretinoin, Idarubicin, Mitoxantrone, and Mercaptopurine), which was completed in January 2015. Before and after the chemotherapy, the patient remained cardiac symptom-free. A series of transthoracic echocardiographies (TTEs) that evaluated cardiac function in April 2012, June 2012, March 2015, and March 2017 revealed normal left ventricular function with an LVEF of more than 55%.

Ten years later, in January 2022, the patient’s mammogram revealed two masses (1.2 cm & 0.6 cm) on the right breast. In March 2022, the patient underwent a breast lumpectomy and sentinel lymph node biopsy. The pathology report revealed invasive ductal carcinoma grade 3 and ductal carcinoma in situ with estrogen receptor-positive (90-95%), progesterone receptor-negative (0%), and HER2-positive (3+). The cancer was staged clinically as stage IA (cT1c, cN0, cM0).

The patient was first treated with paclitaxel (80 mg/m2) plus trastuzumab (2 mg/kg) every week for 12 cycles from June to September 2022, followed by four sessions of radiotherapy (total accumulated dose 5656cGy). The patient’s cardiac function was evaluated by TTE every three months from the initiation of treatment. At the beginning of therapy, the LVEF assessed by TTE shows LVEF 55-60% with normal LV diastolic function. However, the global longitudinal strain equals -14. Repeated echocardiography in September shows LVEF 60-65% with a moderately dilated left atrium. In November, the patient started a nine-month course of trastuzumab (6 mg/kg) as maintenance therapy.

**Figure 1:**
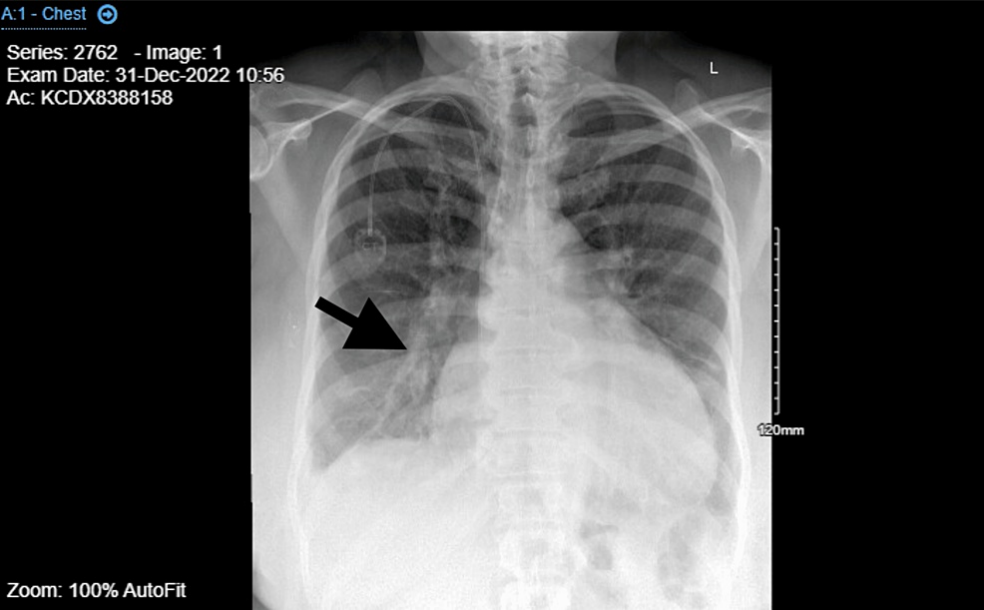
Chest X-ray on 12/5/2023 revealing severe pulmonary edema

One week after the first maintenance dose of trastuzumab infusion (December 2022), the patient presented to the ED with complaints of progressive dyspnea, orthopnea, and bilateral lower extremities swelling for one day. Physical exam showed bilateral bi-basilar crackles and two plus lower extremities pitting edema. EKG showed sinus tachycardia with low-voltage QRS. Troponin I was negative. proBNP was 2035. Chest X-ray showed bilateral pulmonary vascular congestion. Figure 1 shows an enlarged cardiothoracic ratio, increased opacification of pulmonary parenchyma, and enlarged pulmonary arteries as the black arrow indicates.

**Figure 2:**
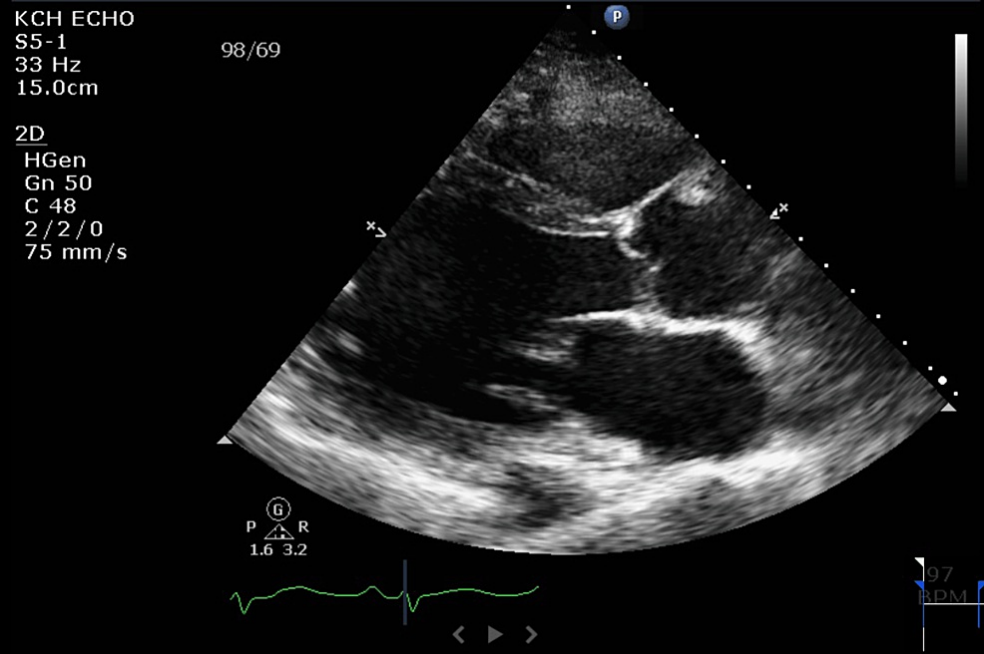
TTE on 12/6/2022 showing the end-systole phase with LVEF 5% TTE: transthoracic echocardiography; LVEF: left ventricular ejection fraction

**Figure 3:**
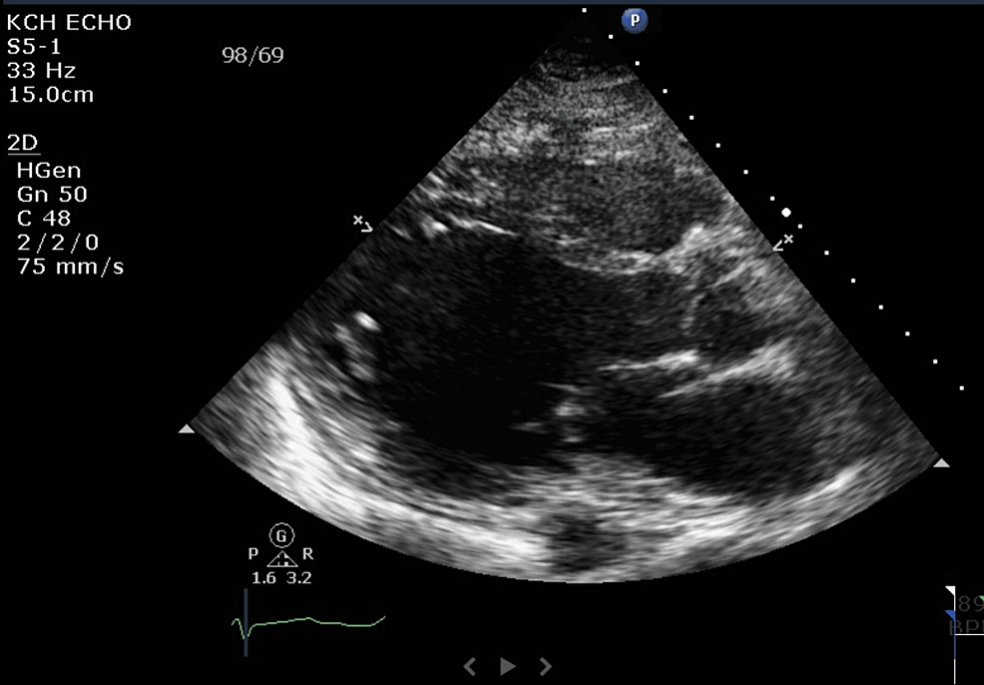
TTE on 12/6/2022 showing the end-diastole phase with LVEF 5% TTE: transthoracic echocardiography; LVEF: left ventricular ejection fraction

After diagnosing acute pulmonary edema, intravenous furosemide was administered, and low-dose Entresto was started. The TTE showed severe diffuse left ventricular hypokinesis and moderately dilated left ventricle with LVEF 5% (Figures 2-3 show enlarged LV chamber size and decreased LV wall changes during systole).

On the next day, the patient’s symptoms improved with intravenous furosemide.

The patient’s history was reviewed carefully and more cardiac workups were completed to assess the possibilities of alternative etiologies. This includes the absence of flu-like symptoms or sick contacts for viral myocarditis; absence of recent travel for Chagas disease; negative urine toxicity test, minimal alcohol use, and the absence of other cardiac toxicity medication use for substance-induced cardiomyopathy; absence of infection, positive blood culture or new-onset valvular regurgitation in TTE for endocarditis; nuclear stress test and percutaneous coronary intervention (PCI) revealed negative for ischemic cardiomyopathy. Therefore, a diagnosis of trastuzumab-induced cardiotoxicity was made, and trastuzumab was discontinued.

**Figure 4:**
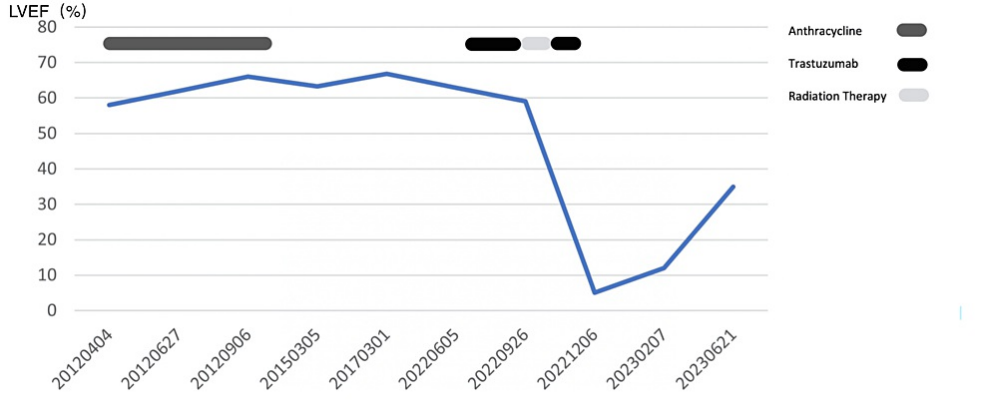
Timeline for LVEF trend LVEF: left ventricular ejection fraction

Given the patient’s baseline soft blood pressure (systolic blood pressure around 90-100s), the patient was discharged with oral furosemide 40 mg daily for congestive heart failure (CHF) symptom control. Sacubitril/valsartan 24-26 mg twice daily, metoprolol succinate 50 mg daily, and dapagliflozin were also started per guideline-directed medical therapy (GDMT). Spironolactone was added in an outpatient follow-up visit in February 2023. Attempts were made to increase sacubitril/valsartan to 49/51 mg twice daily in February and March. However, the patient could not tolerate it and developed symptomatic hypotension. Over a six-month interval follow-up, the patient reported that symptoms had improved gradually. By March 2023, the patient reported dyspnea after walking one block and intermittent lower extremities swelling. By July 2023, the patient was able to walk two to three blocks without dyspnea and denied orthopnea or lower extremities swelling. Repeated TTE in February 2023 showed severe diffuse left ventricular hypokinesis with LVEF of 10%, and another one in July 2023 showed severe diffuse left ventricular hypokinesis with mildly right ventricular hypokinesis, LVEF of 33%. Figure 4 shows the LVEF timeline for the patient. Given the patient’s ongoing LVEF <35% with New York Heart Association (NYHA) II symptoms despite maximally tolerated GDMT, the patient was referred to an advanced heart failure clinic.

## Discussion

We report the rare case of a female patient previously exposed to anthracycline 10 years ago with remaining normal cardiac function. She has now developed acute decompensated heart failure with reduced ejection fraction shortly after receiving trastuzumab for her newly diagnosed early-stage breast cancer. To the best of our knowledge, no previous detailed case reports have described the same clinical scenario as in this case.

Differential diagnosis 

On admission, although trastuzumab-induced cardiotoxicity was at the top of our differential diagnosis, more common etiologies were initially considered for the following reasons. First, well-known risk factors for trastuzumab-induced cardiotoxicity include age over 65 years, obesity, metabolic syndrome, pre-existing cardiovascular disease, and previous anthracycline use - only the last one was presented in our patient [4]. Second, trastuzumab-induced cardiotoxicity present as new onset acute decompensated heart failure is rare: the reported incidence of heart failure with NYHA class III or IV is between 0 and 4% [2]. Third, although the patient had previous exposure to anthracycline, the treatment was completed over 10 years ago without either clinical or image signs of cardiac injury. Diagnostic procedures, including cardiac MRI and endomyocardial biopsy, were offered, however, the patient refused. Upon detailed review of history, absence of other cardiovascular risk factors, and with the support of further laboratory and image studies, we excluded infectious, substance-induced, or ischemic cardiomyopathy as the potential causes in our patient.

It's worth noting that radiation therapy-induced cardiotoxicity was a possibility in our patient. However, there is usually a long delay between radiation exposure and clinical manifestation, and radiation-induced cardiotoxicity is more commonly present as pericardial disease or diastolic dysfunction [5]. Receiving right-side breast radiation therapy, developing symptoms shortly after radiation therapy(within two months), absence of valvular or pericardium changes, and normal diastolic function all made radiation therapy-induced cardiotoxicity less likely. Therefore, our presumed diagnosis was trastuzumab-induced cardiotoxicity in the setting of previous idarubicin exposure 10 years ago.

Trastuzumab-induced cardiotoxicity 

As we discussed earlier, trastuzumab-induced cardiotoxicity rarely presents as new-onset decompensated heart failure in a patient who does not have other comorbidities or risk factors. In retrospection of the initial cardiovascular workups, we found two possible explanations for this rare, dramatic LVEF decline after trastuzumab exposure.

Although the baseline LVEF was 60% in our patient, the pre-treatment left ventricular global longitudinal strain (LV GLS) was -14%, indicating an underlying subclinical cardiac injury that was possibly related to previous anthracycline exposure, which put our patient at particular risk of trastuzumab-related cardiotoxicity. The initial anthracycline treatment left its cardiotoxic effect on the heart as a “first attack” sub-clinically over the past 10 years, evidenced by the preserved LVEF and impaired LV GLS. The preserved LVEF results from substantial cardiac reserves and the activation of cardiac compensatory mechanisms. The patient remained asymptomatic for the past 10 years with gradual exhaustion of the mechanism until she experienced a “second attack” brought by trastuzumab therapy, which incurred additional stress factors and broke the compensation balance [6].

Another possible explanation is the high cumulative dose of the anthracycline (400 mg/m^2^ doxorubicin equivalent) our patient had previously received. In the HERA trial, patients with trastuzumab-associated cardiac dysfunction were found to receive higher cumulative doses of doxorubicin (287 mg/m^2^ v 257 mg/m^2^) before the anti-HER2 therapy [7]. Current guidelines suggest that a shorter time interval between anthracycline and trastuzumab therapy is one of the risk factors for trastuzumab-induced cardiotoxicity. Although it is still unknown if there exists a “safe interval” for the initiation of anti-HER2 therapy after previous anthracycline exposure, a three-month interval was observed to have an incidence of cardiotoxicity that was almost as low as the incidence for those who had not been treated with prior anthracyclines [4]. Unfortunately, our patient still demonstrated clinical HF with a significant LVEF drop 10 years after anthracycline exposure, which we considered long enough to start anti-HER2 therapy safely. As a result, we speculated that the previous cumulative dose of anthracycline may contribute to post-trastuzumab cardiotoxicity independent of the time interval of the treatment.

Pre-chemotherapy risk assessment 

The 2022 ESC Cardio-oncology guidelines recommended the use of the HFA-ICOS Cardio-Oncology cardiovascular risk assessment tool for pre-treatment risk assessment. Based on the type of chemotherapy, pre-treatment cardiac biomarkers, patient’s age, pre-existing cardiovascular risk factors, previous cardio-toxic treatment, and lifestyle risk factors, it classifies patients as low, medium, high, and very high risk and determines if the patient requires a closer monitor or cardio-oncology referral. Before receiving trastuzumab, the patient’s cardiotoxicity risk was classified as low risk, which was identified as safe to initiate trastuzumab without other intervention, and only three-month interval surveillance was required. One limitation of this assessment tool is that it only considers the previous cardio-toxic exposure (anthracycline in this case) but also does not consider the cumulative dose. Another limitation is that it fails to consider a baseline GLS (-14 in this case), which may indicate there is a subclinical cardiac injury before showing any LVEF changes in the TTE. Therefore, a more comprehensive cardiotoxicity risk assessment tool is warranted. To prevent similar unwanted outcomes in prospective patients, before initiating trastuzumab therapy, it is worth assessing the cardiac risk factor more carefully and deciding if further cardiac workup, an early cardio-oncology referral, or even initiating a cardioprotective agent (beta-blockers and/or angiotensin antagonist) [8] is needed. It is also important to adhere to LVEF monitoring every three months strictly. Given the fact that, like our patient, unwanted outcomes can still occur even after taking all the preliminary measures, patients could also benefit from frequent counseling, education, and palliative care for emotional support about this black box warning of cardiomyopathy before and during treatment.

Treatment and symptom response

According to the 2022 AHA guideline, GDMT, which includes sodium-glucose cotransporter-2 Inhibitors (SGLT2i), renin-angiotensin system inhibitors, beta-blockers, and mineralocorticoid receptor antagonists (MRAs), is recommended in patients with heart failure with reduced ejection fraction (HFrEF) to reduce hospitalization and mortality. After elucidating heart failure with an LVEF of 5%, GDMT was initiated per guideline over a period of six months, with clinical improvement evidenced by improved symptoms and LVEF during serial follow-ups. On discharge, our patient was classified as NYHA class IV, and gradually improved to NYHA class III on the three-month follow-up and eventually improved to NYHA class I-II by the time of the six-month follow-up.

The patient’s LVEF dropped from 60% to 5% on admission. We believed this absolute LVEF drop of 55% contained two components, anthracycline and trastuzumab. Over the six-month GDMT therapy, the patient’s LVEF improved from 5% to 33%. Given that anthracycline-induced cardiac injury is irreversible, we believe this portion of LVEF recovery (from 5% to 33%) is due to the reversible component of trastuzumab cardiotoxicity. However, we observed a longer recovery time in our patient, compared to the larger database. Studies show that in patients with trastuzumab-induced systolic dysfunction, 84% of the patient’s LVEF had returned to baseline (0.5+/- 0.11) after trastuzumab was discontinued. The mean time to recovery of LVEF was 1.5 months [9]. We believe that the delayed recovery in our patient is due to trastuzumab cardiotoxicity superimposing anthracycline toxicity, and a slow introduction (over six months) of GDMT might also play a role. Since the other portion of the LVEF drop is due to the irreversible component of anthracycline cardiotoxicity, it is difficult to predict to what level the LVEF will improve because the relative proportion of the two components is unclear.

This uncertainty of the reversibility of our patient’s cardiac function recovery raises another question regarding the timeline to consider implantable cardioverter defibrillator (ICD) implantation. Although it is widely accepted that the critical waiting period is three months for ischemic cardiomyopathy, there is no clear guideline for chemotherapy-induced new-onset HF. Given the reversible potential of trastuzumab-induced cardiomyopathy, possible delayed optimization of GDMT in our patient, and a borderline LVEF (33%), we predict that the patient’s cardiac function will continue to recover over a period of time. To avoid complications such as infection, inappropriate shocks, or even anxiety or depression [10-11], we decided to postpone ICD implantation and monitor TTE for an extended period of one year.

Lastly, trastuzumab treatment interruption may lead to worse cancer outcomes, such as disease recurrence or a worsening overall cancer prognosis. A large population study that enrolled 5547 patients shows that compared to patients who completed 12-month trastuzumab therapy, patients who failed to complete 12-month trastuzumab therapy had higher breast cancer-specific mortality within five years (2.7% vs 9.2%-10%) [12]. Since trastuzumab was discontinued due to our patient’s severe symptomatic LV dysfunction, it is unclear how the alteration in cancer treatment will change the outcome of her breast cancer. More investigation regarding the impact of trastuzumab interruption on early breast cancer treatment outpatient is needed for future research and clinical practice.

Limitation and future avenues

There are some limitations of our study. A cardiovascular magnetic resonance (CMR) or myocardial biopsy was necessary to confirm the diagnosis of trastuzumab-induced cardiotoxicity [13]. A myocardial biopsy can also guide us to understand the possible cardiac injury from trastuzumab and anthracycline and thus predict our patient’s cardiac function recovery potential. Although CMR and myocardial biopsy were offered, our patient declined. Another limitation was the GLS was only calculated on the TTE before the patient received trastuzumab. The lack of GLS in the repeat TTE by the completion of the induction cycle of trastuzumab therapy might lead to a failure to detect early cardiac damage. Studies show that there are some specific biomarkers such as high-sensitivity troponin, N-terminal pro-B-type natriuretic peptide (NT pro-BNP), myeloperoxidase (MPO), C-reactive protein (CPR), and suppressor of tumorigenicity 2 (ST2), might serve as early detection of trastuzumab and anthracycline-induced cardiotoxicity and might be potential avenues for future research [14].

## Conclusions

This unique case elaborates on a rare, catastrophic cardiac toxicity effect of trastuzumab in a young patient who had no other cardiovascular risk but had remote exposure to anthracycline. As a result, trastuzumab therapy had to be terminated permanently. To summarize, our case highlights the limitation of a commonly used prechemotherapy cardiovascular risk assessment tool and the lack of a clear guideline for ICD implantation in patients with trastuzumab-induced HFrEF. Future studies are warranted to refine pre-chemotherapy cardiovascular risk stratification tools, establish a clear guideline for chemotherapy-induced HFrEF management, and investigate the impact of trastuzumab interruption on cancer treatment outcomes.

## References

[1] Zamorano JL, Lancellotti P, Rodriguez Muñoz D (2016). 2016 ESC Position Paper on cancer treatments and cardiovascular toxicity developed under the auspices of the ESC Committee for Practice Guidelines: the Task Force for cancer treatments and cardiovascular toxicity of the European Society of Cardiology (ESC). Eur Heart J.

[2] Hamirani Y, Fanous I, Kramer CM, Wong A, Salerno M, Dillon P (2016). Anthracycline- and trastuzumab-induced cardiotoxicity: a retrospective study. Med Oncol.

[3] Ewer MS, Ewer SM (2010). Cardiotoxicity of anticancer treatments: what the cardiologist needs to know. Nat Rev Cardiol.

[4] Onitilo AA, Engel JM, Stankowski RV (2014). Cardiovascular toxicity associated with adjuvant trastuzumab therapy: prevalence, patient characteristics, and risk factors. Ther Adv Drug Saf.

[5] Belzile-Dugas E, Eisenberg MJ (2021). Radiation-induced cardiovascular disease: review of an underrecognized pathology. J Am Heart Assoc.

[6] Suter TM, Ewer MS (2013). Cancer drugs and the heart: importance and management. Eur Heart J.

[7] Suter TM, Procter M, van Veldhuisen DJ (2007). Trastuzumab-associated cardiac adverse effects in the herceptin adjuvant trial. J Clin Oncol.

[8] Omland T, Heck SL, Gulati G (2022). The role of cardioprotection in cancer therapy cardiotoxicity: JACC: CardioOncology state-of-the-art review. JACC CardioOncol.

[9] Ewer MS, Vooletich MT, Durand JB, Woods ML, Davis JR, Valero V, Lenihan DJ (2005). Reversibility of trastuzumab-related cardiotoxicity: new insights based on clinical course and response to medical treatment. J Clin Oncol.

[10] Dichtl W, Wolber T, Paoli U (2011). Appropriate therapy but not inappropriate shocks predict survival in implantable cardioverter defibrillator patients. Clin Cardiol.

[11] Sears SF, Todaro JF, Urizar G (2000). Assessing the psychosocial impact of the ICD: a national survey of implantable cardioverter defibrillator health care providers. Pacing Clin Electrophysiol.

[12] Rushton M, Lima I, Tuna M (2020). Impact of stopping trastuzumab in early breast cancer: a population-based study in Ontario, Canada. J Natl Cancer Inst.

[13] Bouwer NI, Jager A, Liesting C (2020). Cardiac monitoring in HER2-positive patients on trastuzumab treatment: A review and implications for clinical practice. Breast.

[14] Jiang J, Liu B, Hothi SS (2022). Herceptin-mediated cardiotoxicity: assessment by cardiovascular magnetic resonance. Cardiol Res Pract.

